# Supplementary material for: Neutrophil Extracellular Traps Mediate Bovine Endometrial Epithelial Cell Pyroptosis in Dairy Cows with Endometritis
Source: Int J Mol Sci. 2022 Nov 13;23(22):14013. doi: 10.3390/ijms232214013 (PMC9694523; doi:10.3390/ijms232214013)
Supplement: Supplementary file 1 [file ijms-23-14013-s001.zip › ijms-1932922-supplementary.pdf]

Supplementary Figures

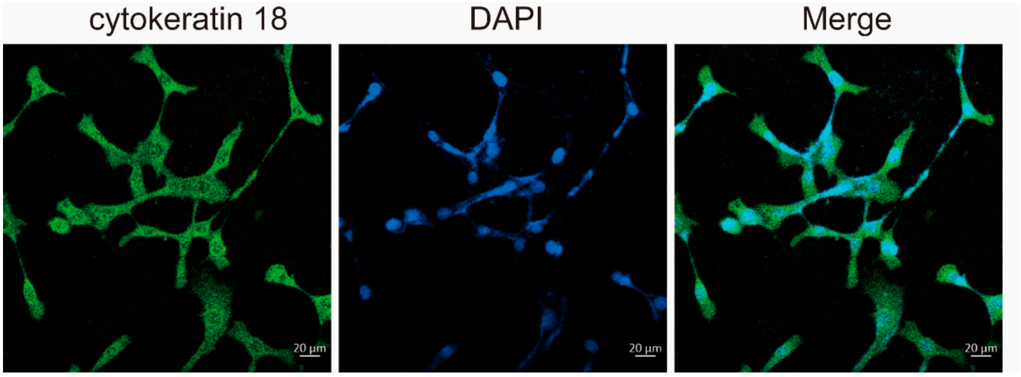

Supplementary Figure S1. the morphology of primary BEECs isolated from cow endometrial tissue that was stained by cytokeratin 18 antibody. White bar = 20 µm.

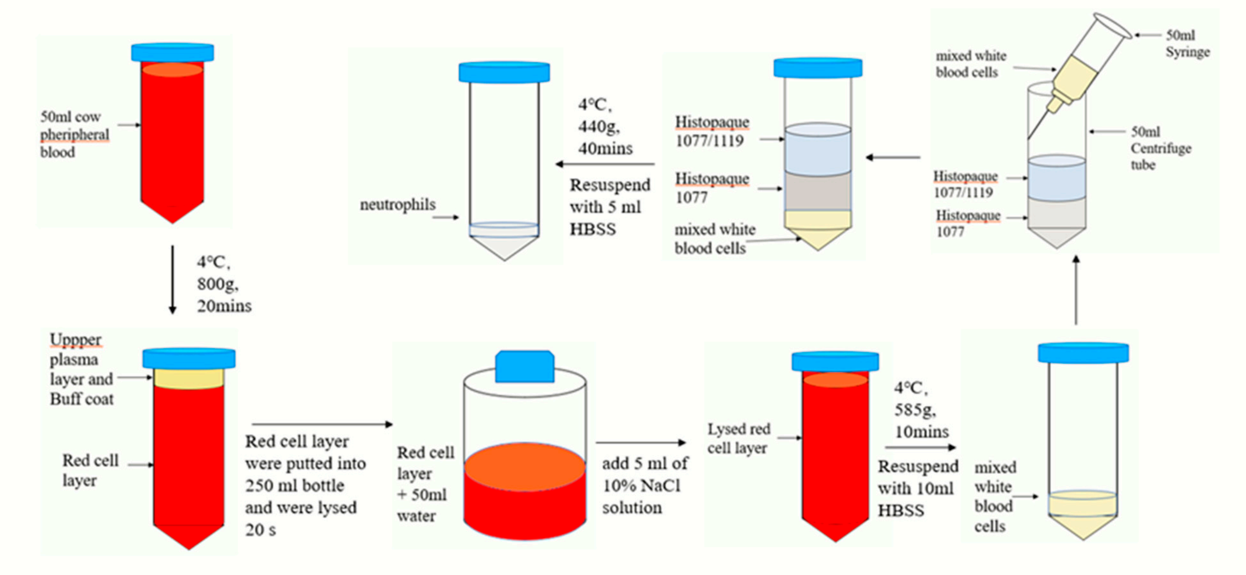

Supplementary Figure S2. Flow chart of the isolation of neutrophils from the peripheral blood of dairy cows

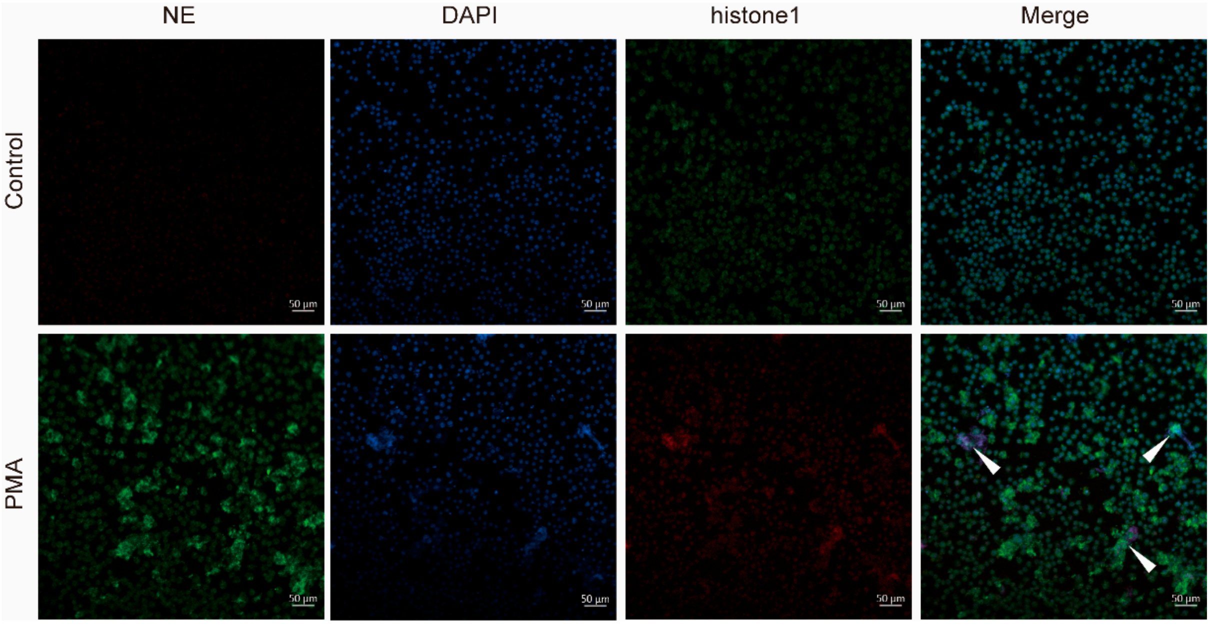

Supplementary Figure S3. Phorbol 12-myristate 13-acetate (PMA) induced NETs formation from neutrophils isolated from cow peripheral blood. White arrow indicates NETs. White bar = 50 μm.

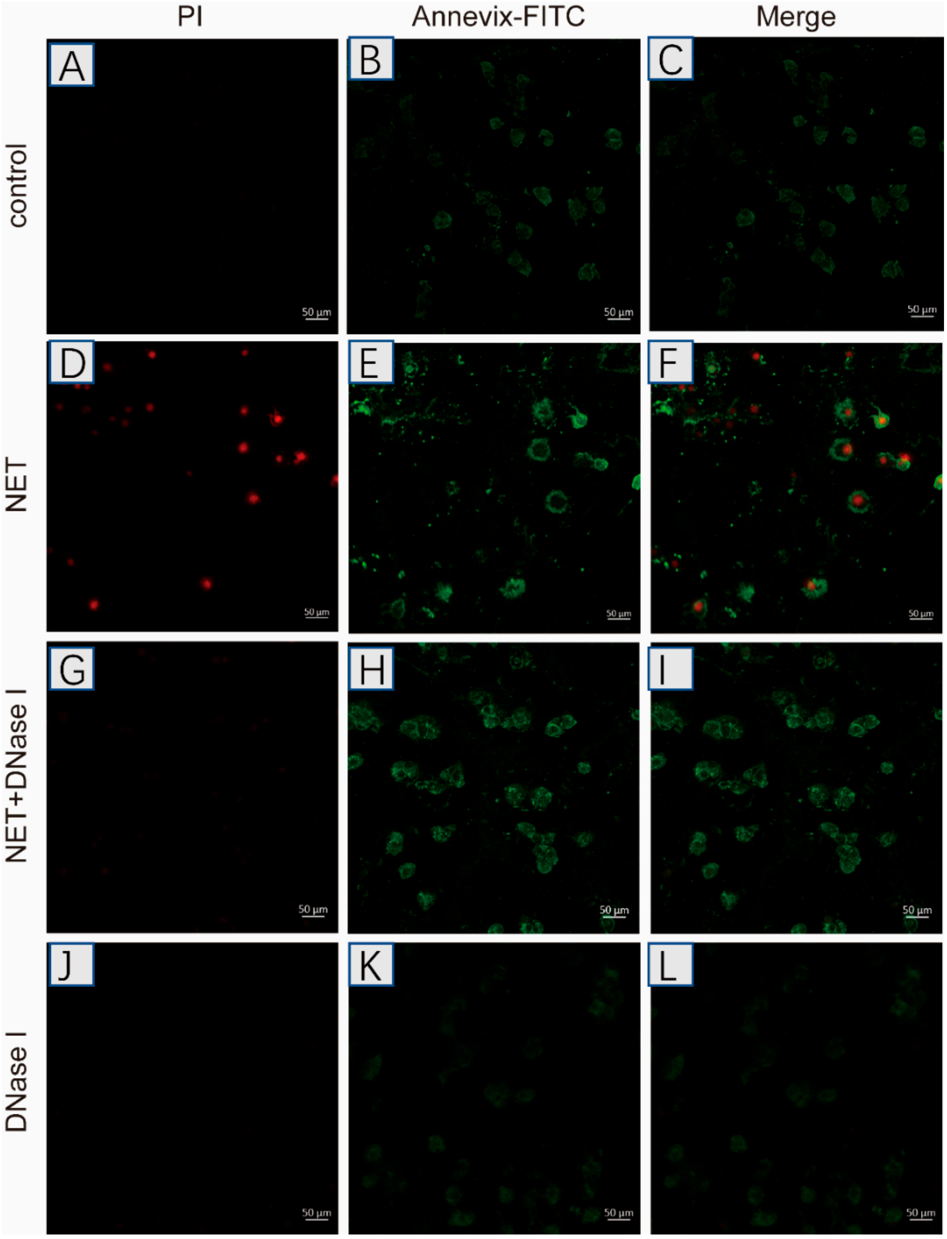

Supplementary Figure S4. Confocal microscopy for the same cells stained with propidium iodide (PI) (red) and Annevix-FITC(green) to detect nuclear shape and plasm membrane in BEECs. White bar = 50 μm.
